# Single-drill implant induces bone corticalization during submerged healing: an in vivo pilot study

**DOI:** 10.1186/s40729-019-0198-y

**Published:** 2020-01-15

**Authors:** Paolo Trisi, Antonello Falco, Marco Berardini

**Affiliations:** Biomaterial Clinical and Histological Research Association, Private Practice, Via Galilei 8, 65122 Pescara, Italy

**Keywords:** Dental implant, Osseocorticalization, Osseointegration, Low-density bone, Bone histology

## Abstract

**Purpose:**

The aim of the present paper is to evaluate a simplified implant site preparation technique to preserve bone bulk and enhance osseointegration using a new conical self-tapping implant in cancellous bone.

**Materials and methods:**

Ten Expander® 3.8 × 10 mm implants (NoDrill®, Milano, Italy) were inserted in the right side (test group) of sheep’s iliac crest using only the pilot drill 1.8 mm in diameter. Ten 3.8 × 10 mm Dynamix® implants (Cortex, Shlomi, Israel) were inserted in the right side (control group) of the same animals following the drilling protocol provided by the manufacturer. Histological, histomorphometric, and biomechanical analyses were performed after 2 months.

**Results:**

Implants that belonged to the test group showed a %BIC of 70.91 ± 7.95 while the control group implants had a %BIC value of 49.33 ± 10.73. The %BV was 41.83 ± 6.30 in the test group and 29.61 ± 5.05 in the control group. These differences were statistically significant. A phenomenon of osseocorticalization, characterized by more bone volume percentage around implant area than in the neighboring areas, caused by implant threads geometry, was evident in the test group.

**Conclusion:**

This surgical protocol allows to insert an innovative fixture geometry in low-density bone using only a pilot drill. This technique demonstrated many clinical and histological advantages with respect to standard implant drilling procedures and classical implant geometry.

## Introduction

It is well documented that implant initial bone fixation, known as primary implant stability, represents the pre-requisite to achieve a successful long-term osseointegration [1].

Many studies demonstrated that the implant primary stability is strictly influenced by host bone density [2], fixture geometry [3, 4], and surgical technique used for preparing bone implant bed [5]. Other studies [6, 7] highlighted that host bone quality and fixture macro geometry as main factors able to influence the primary implant stability.

The implant bone site preparation plays a key role in osseointegration development because it allows to obtain an implant bone bed suitable for the fixture dimensions ensuring primary implant stability.

The excessive surgical trauma prior to implant insertion and the bone temperature rise during standard drilling procedures [8] are other crucial factors modifiable by the surgeon, whose importance is often underestimated.

A minimally traumatic bone drilling is strongly recommended to preserve much bone tissue as possible without impairing its healing potential [9].

To this end, several surgical techniques [10] have been proposed to avoid or reduce bone sacrifice during implant placement procedures to enhance primary implant stability and bone quality.

Some authors suggested to undersize the osteotomic implant site with respect to the implant diameter of about 10% in order to reduce bone cutting and enhance primary implant stability [11, 12].

An alternative to implant drilling procedures is represented by the osteotome technique [13] that aimed to compact the bone with the mechanical action of cylindrical steel instruments along the osteotomic walls. This procedure increases the clinical implant success in poor bone density [14] although fractured trabeculae and debris could cause a delay in osseointegration process [15, 16].

The osseodensification (OD) technique, recently introduced by Huwais et al. [17], used special burs in non-cutting rotation mode in order to move bone inside the osteotomic site instead of removing it. This technique allows to preserve native bone and enhance the bone volume around implants [18] supporting high bone contact with the titanium.

The aim of the present paper is to evaluate a reduced implant site preparation technique to preserve bone bulk and enhance primary stability using a new conical self-tapping implant in cancellous type IV bone. A histologic, histomorphometric, and biomechanical comparative analysis was conducted in low-density bone of the sheep’s iliac crest. The hypothesis to test is if undersized preparation and compressing implants yield a higher BIC and higher torque and ISQ than standard implant inserted with traditional drilling protocols.

## Materials and methods

The Ethics Committee for Animal Research of the Veterinary School of the University of Teramo (Teramo, Italy) approved the study protocol, which followed the guidelines established by the European Union Council Directive of February 2013 (R.D.53/2013).

Two female sheep, 4–5 years old, were included in the study. Clinical examination determined that all animals were in good general health. Exclusion criteria included general contraindications (pregnancy, systemic disease) to implant surgery and active infection, or severe inflammation in the area intended for implant placement.

The animals were given thiopental (Thiopental, Höchst, Austria) for induction of anesthesia as needed. After oro-tracheal intubation and ventilation, anesthesia was sustained with nitrous oxide oxygen with 0.5% halothane. Physiologic saline solution was administered during surgery for fluid replacement.

The edges of the iliac crests were exposed through a skin incision of 15 cm in length. The skin and facial layers were opened and closed separately.

After dissection of the soft tissues, the bone was exposed and five undersized osteotomic sites were prepared in each (left and right) side of the iliac crest. In the right side of each animal (test group), implant bone sites were prepared using only the pilot drill 1.8 mm in diameter. Ten Expander® 3.8 × 10 mm implants (NoDrill®, Milano, Italy) were inserted in the right side of both animals with a hand control wrench. Maximum insertion torque values were between 45 and 60 N/cm. In the left side of each animal (control group), implant bone sites were prepared using the following burs sequence: pilot drill 1.8 mm in diameter, twist drill 2.8 mm in diameter, and the final drill 3.2 mm in diameter. Ten 3.8 × 10 mm Dynamix® implants (Cortex, Shlomi, Israel) were inserted in the left sides of both animals. Maximum insertion torque values were between 30 and 45 N/cm. The implant drilling procedures were carried out under profuse saline irrigation (1000 rpm). Implants were inserted in cancellous type IV bone.

After implant insertion, cover screws were secured and the surgical wounds were closed by a resorbable periosteal-muscular inner suture, followed by an external cutaneous 2-0 silk suture.

Each animal underwent systemic antibiotic therapy for 5 days with 8 ml long-acting Clamoxil (Pfizer Limited, Sandwich, USA). After surgery, animals received appropriate veterinary care and were allowed free access to water and standard laboratory nutritional support throughout the trial period.

The sheep were sacrificed 2 months after implantation by an overdose of sodium thiopental (Thiopental, Höchst, Austria).

### Micromotion analysis

Bone blocks containing the implants were retrieved from each side of the iliac crest. Each implant was fitted with a one-piece 11-mm straight abutment.

The bone blocks were fixed on a customized loading device to measure implant secondary stability according to a previously described technique [19]. A digital force gauge (Akku Force Cadet, Ametek, Largo, USA) and, on the opposite side, a digital micrometer (Mitutoyo Digimatic Micrometer, Kawasaki, Japan) were used to measure implant micromotion during load application. Horizontal forces of 25 N/cm were applied onto the abutment of the implant perpendicularly to the major axis, and the lateral displacement was measured by the digital micrometer 10 mm above the crest. This parameter represents the “value of the actual micromotion” (VAM) as previously published [20] and validated [21].

### ISQ analysis

Resonance frequency analysis was assessed at the time of animal sacrifice (after 2 months of healing) with the latest Ostell device (Osstell AB, Göteborg, Sweden). The implant stability quotient (ISQ), which ranged between 0 and 100, was recorded.

### Removal torque value (RTV) testing

Removal torque value (RTV) was measured at the time of animal sacrifice (2 months after implantation) after VAM measuring procedures. The RTV was evaluated and recorded for each implant using a digital hand-operated torque wrench (Tonichi STC400CN) by unscrewing the implants until interfacial failure occurred. The digital torque wrench automatically registered the peak removal torque value on the digital display. After the initial interface detachment, the implants were repositioned back in their initial position as accurately as possible and processed for histologic analysis. Although the interfacial detachment created an artifact at the interface, its analysis would still be reliable according to Sennerby et al. [22], who used a similar procedure to study the morphology of the bone-metal rupture.

### Histomorphometric analysis

Specimens were immediately fixed in 10% neutral buffered formalin and processed for histologic analysis. After dehydration, samples were infiltrated with a methyl-methacrylate resin from a starting solution 50% ethanol/resin and subsequently 100% resin, with each step lasting 24 h. After polymerization, the blocks were sectioned and then ground down to about 40 μm. Toluidine blue staining was used to analyze the different ages and remodeling pattern of the bone. The histomorphometric analysis was performed by digitizing the images from the microscope via a JVC TK-C1380 Color Video Camera (JVC Victor Company, Yokohama, Japan) and a frame grabber. The images were acquired with a × 10 objective over the entire implant surface. Subsequently, the digitized images were analyzed by the image analysis software IAS 2000 (Delta Sistemi, Roma, Italy).

For each section, the two most central sections were analyzed and morphometrically measured. The histomorphometric parameters calculated were the % bone-to-implant contact (%BIC) and the bone volume (%BV).

### Host bone density analysis

In both iliac crests of each animal, a bone sample was harvested close to the implant sites. The bone samples collected were analyzed in order to establish the bone volume percentage (basal %BV).

### Statistical analysis

Biomechanical (VAM, RT, and ISQ) and histomorphometrical data (BIC% and BV%) of test and control groups were statistically compared by the *T* test using a dedicated software (GraphPad Prism 6 - www.graphpad.com).

## Results

No implant failure was observed after 2 months of healing. The clinical examination, done immediately after the bone block retrieval, showed no crestal bone resorption. No bone defects around implants, such as fenestration or dehiscence, were detected. The host bone density expressed in bone volume percentage (basal %BV) was 26.17 ± 2.35. This low value of BV% is common in soft bone, according to the NHS bone classification [23].

**Figure 1:**
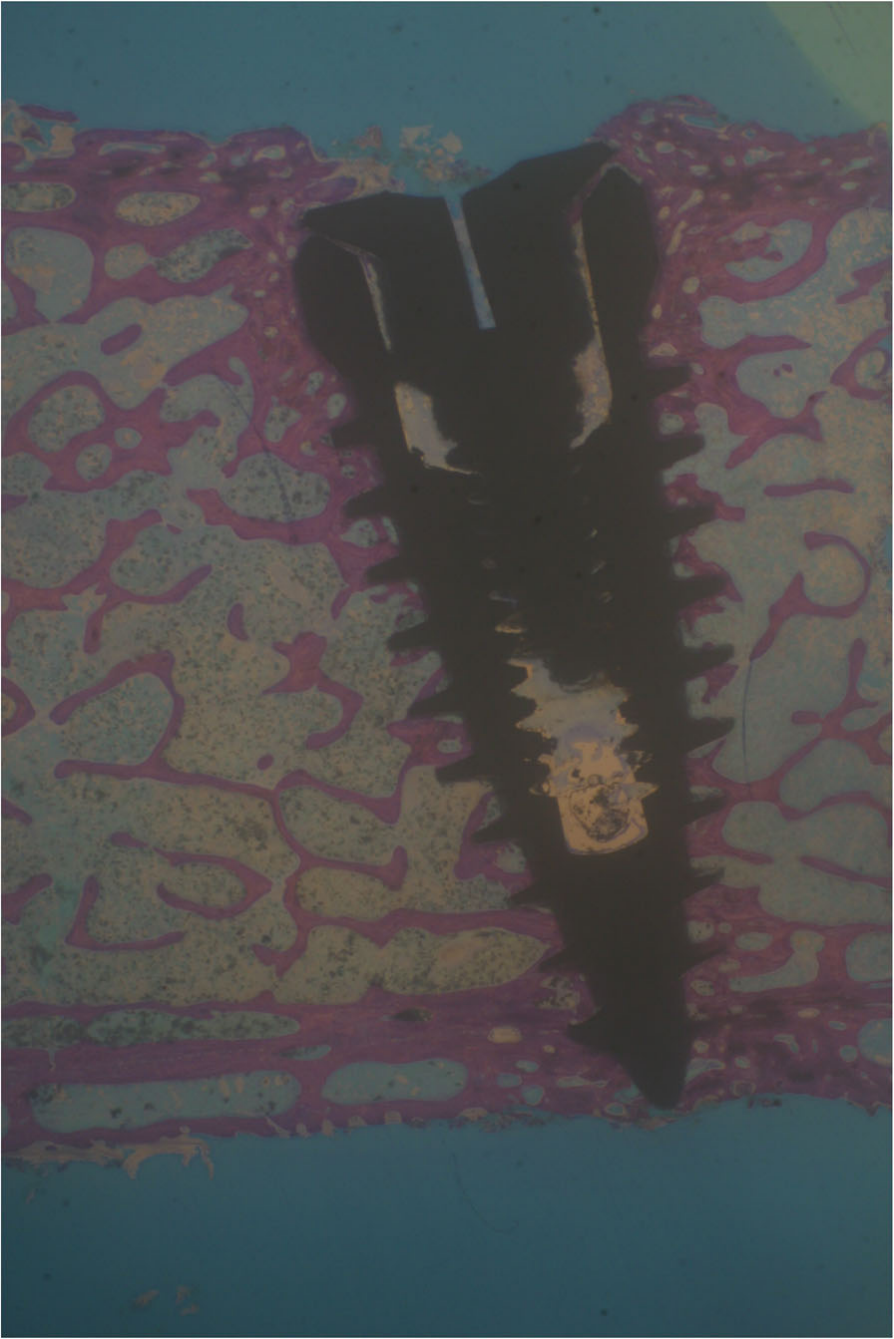
Test group. The implant achieved a high osseointegration degree. The newly formed bone appeared well interconnected with the pre-existing trabeculae. The “corticalization” phenomenon is evident: the bone appears densified around a titanium implant (magnification × 8—toluidine blue)

**Figure 2:**
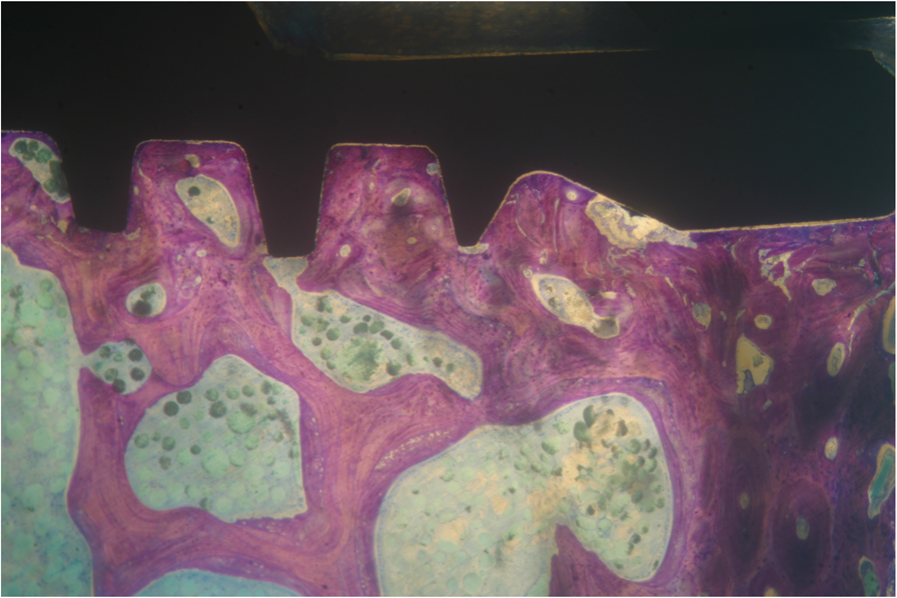
Test group. Implants in the test group showed an extremely high percentage of bone directly contacted to implant surface (magnification × 25—toluidine blue)

**Figure 3:**
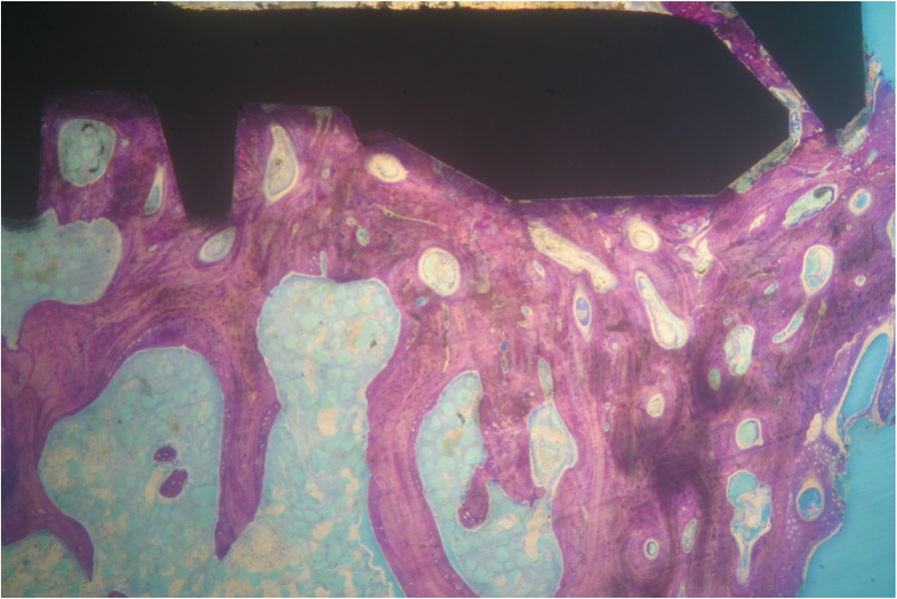
Test group. The present histological photo showed a continuous thin layer of newly formed bone along the neck area of the implant (magnification × 25—toluidine blue)

**Figure 4:**
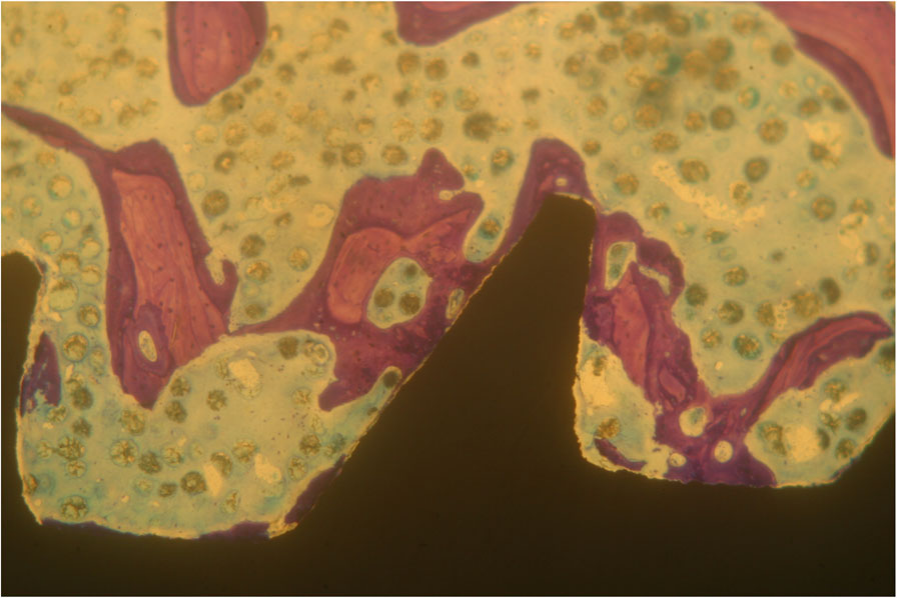
Control group. No bone condensation was possible with traditional burs and standard implant (magnification × 25—toluidine blue)

**Figure 5:**
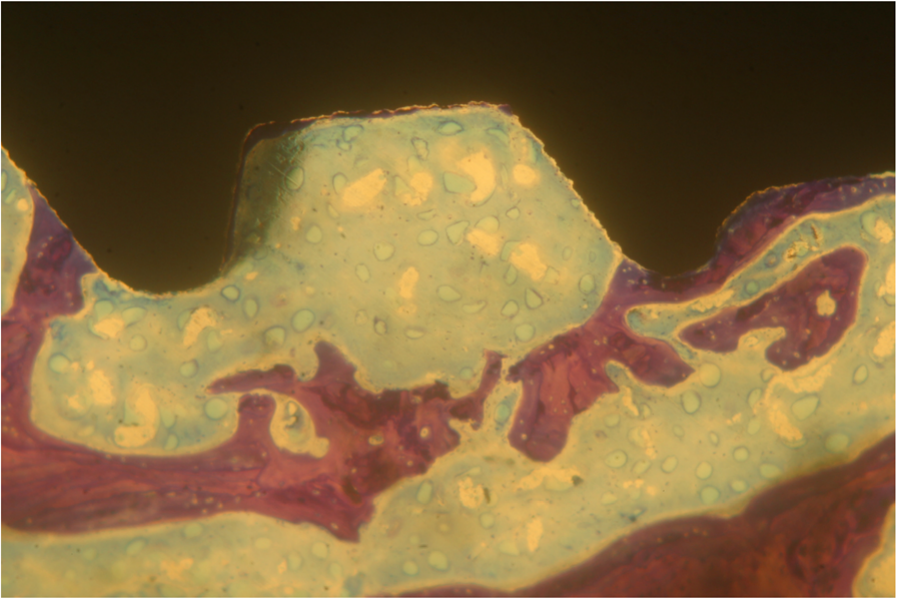
Control group. Implants belonging to the control group showed some small surface areas not contacted with bone (magnification × 25—toluidine blue)

**Figure 6:**
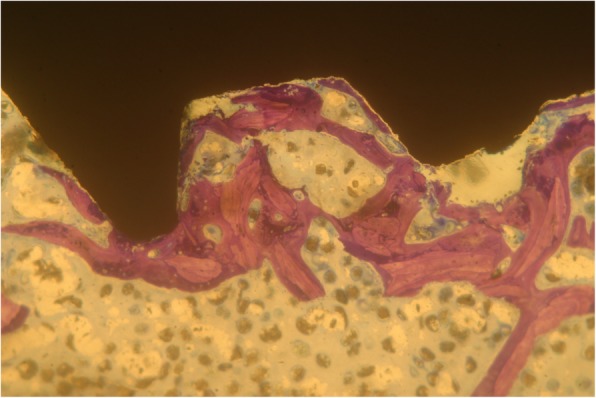
Control group. Some implant thread areas were not covered by bone layer (magnification × 25—toluidine blue)

Implants that belonged to the test group showed a bone to implant contact percentage (%BIC) of 70.91 ± 7.95 while the control group implants had a %BIC value of 49.33 ± 10.73. The %BV was 41.83 ± 6.30 in the test group and 29.61 ± 5.05 in the control group (Figs. 1, 2, 3, 4, 5, and 6). These histomorphometric parameters were statistically different between the two groups. The statistical comparison between the host bone density (basal %BV) and %BV in the test group revealed that expander® implants were able to increase in a significant way the host bone density (Tables 1, 2 and 3).

**Table 1:** Basal bone volume percentage (basal %BV) was compared to %BV around implants after 2 months of healing in both groups. %BV in the test group was significantly higher than basal %BV (*P* < 0.05)

Basal %BV 26.17 ± 2.35	Test group	Control group
	41.83 ± 6.30*	29.61 ± 5.05

*Significant

**Table 2:** Mean values of histomorphometric parameters (%BIC and %BV) and biomechanical values (VAM, reverse torque, and ISQ) of each implant group

Implant type	BIC%	BV%	Vam (μm) ± SD	Reverse torque (N/cm) ± SD	ISQ value ± SD
Test group	70.91 ± 7.95	41.83 ± 6.30	82.6 ± 23.27	98.2 ± 16.81	63.5 ± 1.30
Control group	49.33 ± 10.73	29.61 ± 5.05	60.5 ± 16.58	98.8 ± 24.40	59.4 ± 1.39

**Table 3:** Statistical comparison (*T* test) of examined parameters between the test and control groups. The histomorphometric analysis demonstrated significant differences in BIC% and %BV values between the two implant groups

BIC%	*P* < 0.05
BV%	*P* < 0.05
Vam	*P* < 0.05
Reverse torque	*P* > 0.05 Ns
ISQ	*P* < 0.05

The biomechanical analysis of secondary implant stability revealed a VAM value of 82.6 ± 23.27 in the test group and 60.5 ± 16.58 in the control group. The reverse torque (RT) was 98.2 ± 16.81 in the test group and 98.8 ± 24.40 in the control one. Histomorphometric and biomechanical data of both groups are summarized in Table 2.

Expander implant surface (test group) was covered by a thick layer of newly formed bone induced by the osteoconduction properties of the implant surface. A considerable amount of fractured trabeculae that led to bone chip condensation was present around implant threads. The osseocorticalization phenomenon characterized by more bone volume percentage around the implant area than in the neighboring areas, caused by implant threads geometry, was evident at low magnification. A reparative bone formation process that connected the fractured bone trabeculae to bone fragments and/or to the implant surface was evident, with remodeling phenomena characterized by osteoclastic resorption coupled with osteoid formation.

## Discussion

Results from the present study clearly show that it is possible to insert an implant using a one-step concept for the surgical preparation of the bone bed in cancellous bone.

Guazzi et al. [24], comparing the clinical outcome of implants inserted in sites prepared with a simplified protocol consisting of one large single drill versus multiple conventional drilling steps, demonstrated less surgical time which led to less postoperative morbidity in the test group.

Gehrke et al. [25] failed in finding differences in implant stability (RFA analysis) between implant inserted with a drill sequence or single large drill.

In the present study, the single-drill protocol was essentially different from that used from previous authors because the osteotomic sites resulted in undersized preparation with respect to the implant diameter. In this way, it is possible to keep more bone inside the implant bed with respect to a large final drill. Also, the significant thermal changes highlighted by some studies [26, 27] when only final large drill is used as single step are avoided by the present surgical protocol which involved the use of only one low-diameter bur.

Undersizing the osteotomic site is a well-documented [28, 29] surgical technique that allows to increase primary implant stability in poor bone density. From the biomechanical standpoint, an undersized drilling protocol is demonstrated to be effective in increasing insertion torque in low-density bone [30].

Some authors [31] theorized that a 10% undersized protocol, in poor density bone, is sufficient to improve the primary stability of the implant.

In the present study, the undersized bone preparation of the implant site of about 2 mm compared to a 4-mm-diameter implant (almost 100%) allows to obtain an excellent implant primary stability with insertion torque peaks greater than 45 N/cm. The implant geometry characterized by self-tapping threads helps the implant body to penetrate into the bone trabeculae without creating excessive bone dust that could delay the osseointegration processes. The high percentage of bone-implant contact (% BIC), almost double compared to the initial bone density, demonstrates an effective action of bone compaction.

The comparison between the basal %BV and %BV in the test group revealed that these innovative implants were able to increase in a significant way the peri-implant bone density with respect to starting host bone density. This increased peri-implant bone density extended from about 0.5–0.7 mm beyond the implant perimeter causing an osseocorticalization around the fixture profile.

A recent study [32] confirmed that the use of the single-drill surgical approach is less invasive and could promote osseointegration. In addition, the accuracy of implant site preparation is better when the single step procedure is used.

## Conclusions

The hypothesis was accepted. Compressing implants with single-drill bone preparation demonstrated many clinical and histological advantages with respect with to standard implant drilling procedures and classical implant shape and design in low-density bone type. It is possible to summarize the advantages of this technique as follows: higher bone to implant contact percentage than the control group (due to the innovative fixture geometry that causes bone compaction), speed of execution (only one-step preparation in low-density bone), high primary implant stability (undersized preparation matched to special fixture shape and thread geometry), high manageable final implant position, high patient comfort, and less cost.

The present pilot study involves only two animals; this limitation could influence the statistical observations we have discussed. Future in vivo studies with a bigger animal numbers or clinical studies with a high sample size are needed in order to confirm the results of the present paper.

## Data Availability

All data and materials are available from the corresponding author in Pescara, Italy.
